# Combined Metabolome and Transcriptome Analyses of Maize Leaves Reveal Global Effect of Biochar on Mechanisms Involved in Anti-Herbivory to *Spodoptera frugiperda*

**DOI:** 10.3390/metabo14090498

**Published:** 2024-09-14

**Authors:** Tianjun He, Lin Chen, Yingjun Wu, Jinchao Wang, Quancong Wu, Jiahao Sun, Chaohong Ding, Tianxing Zhou, Limin Chen, Aiwu Jin, Yang Li, Qianggen Zhu

**Affiliations:** 1 College of Ecology, Lishui University, Lishui 323000, China; baiyun12_12@163.com (T.H.); jcw199907@163.com (J.W.); clmit@zju.edu.cn (L.C.); kinaw@zafu.edu.cn (A.J.); 2 Lishui Institute of Agricultural and Forestry Sciences, Lishui 323000, China; fay321@126.com (L.C.); lsqcw@163.com (Q.W.); 2022205014@stu.njau.edu.cn (J.S.); lsdch_74@163.com (C.D.); 13757809816@163.com (T.Z.); 3 Ecological Forestry Development Center of Suichang County, Lishui 323300, China; scxwyj@163.com; 4 Soil Fertilizer and Plant Protection Station of Lishui City, Lishui 323000, China

**Keywords:** anti-herbivory, defense responses, lipids, MAPK signaling, phytohormone signaling, secondary metabolites, terpenoids

## Abstract

Fall armyworm (FAW, *Spodoptera frugiperda*) has now spread to more than 26 Chinese provinces. The government is working with farmers and researchers to find ways to prevent and control this pest. The use of biochar is one of the economic and environmentally friendly strategies to increase plant growth and improve pest resistance. We tested four *v*/*v* combinations of bamboo charcoal with coconut bran [BC1 (10:1), BC2(30:1), BC3(50:1)] against a control (CK) in maize. We found that plant height, stem thickness, fresh weight and chlorophyll content were significantly higher in BC2, in addition to the lowest FAW survival %. We then compared the metabolome and transcriptome profiles of BC2 and CK maize plants under FAW herbivory. Our results show that the levels of flavonoids, amino acids and derivatives, nucleotides and derivatives and most phenolic acids decreased, while terpenoids, organic acids, lipids and defense-related hormones increased in BC-grown maize leaves. Transcriptome sequencing revealed consistent expression profiles of genes enriched in these pathways. We also observed the increased expression of genes related to abscisic acid, jasmonic acid, auxin and MAPK signaling. Based on these observations, we discussed the possible pathways involved in maize against FAW herbivory. We conclude that bamboo charcoal induces anti-herbivory responses in maize leaves.

## 1. Introduction

China ranks second in maize production, supplying 23% of the world market [1]. With an increase of 4.2%, the country will produce 288.8 million tonnes in 2024 and is set to become self-sufficient in maize production in the next six years (https://fas.usda.gov/; accessed on 5 May 2024). However, this is possible with optimal crop management, which is directly linked with crop nutrition, as well as protection from biotic and abiotic stresses. Among the biotic stresses, fall armyworm (FAW, *Spodoptera frugiperda*) is a serious threat to maize production in China. Since it was first reported in 2018 [2], it has now spread to 26 Chinese provinces, prompting the government to initiate several warning and prevention systems [3]. Fall armyworm can cause 20–50% yield losses [4]; therefore, the Chinese government is currently focused on developing strategies to control the spread of FAW. The opted control methods included in “the National Information Platform for the Prevention and Control of the Fall Armyworm” are chemical, physical, biological, and ecological measures.

The most appropriate control strategy is to develop tolerant or resistant varieties. This is a long-term and economically favorable strategy for the farmers, as they do not have to bear the additional cost of insecticides. It is also one of the main pillars of integrated pest management [5]. However, it is important to understand the key defense mechanisms in host plants. When herbivores such as FAW attack the host plant, natural defense responses are activated. These induced defense responses play a very important role in protecting the plant from ongoing and future attacks. Plants have both direct and indirect induced responses against such attacks [6]. They can also recognize insect-induced elicitors upon herbivory and activate signaling cascades (phytohormone [7] and MAPK [8]). These pathways relay signals and further activate defense-related pathways, leading to the increased biosynthesis and accumulation of defensive secondary metabolites [9] and activation of resistance genes [10]. Earlier work on the responses of rice plants to herbivory by FAW has shown the increased expressions of genes related to phytohormone biosynthesis and signaling, reactive oxygen species (ROS) homeostasis, and secondary metabolite biosynthesis [11]. Transcriptome analysis of sugarcane revealed the differential regulation of genes enriched in pathways, including amino acid biosynthesis, flavonoid biosynthesis, linoleic acid metabolism, oxidative phosphorylation, plant–pathogen interaction, alkaloid and terpenoid biosynthesis and hormone signaling [12]. This study also reported the differential accumulation of metabolites related to the mentioned pathways. Like other plants, maize also responds to herbivory by reprogramming its metabolism. However, limited studies reported the global transcriptome and metabolome profiles of maize in response to FAW herbivory [13,14]. Thus, understanding the transcriptional and metabolic reprogramming in maize in response to herbivory by FAW is critical [15]. Such information is highly desirable for maize breeders to develop varieties having higher resistance or tolerance to FAW. 

Earlier research on the host plant responses to FAW attacks has shown that plant hormones such as jasmonic acid (JA), salicylic acid (SA) and others are associated with anti-herbivory responses [12]. For example, in rice, the leaves treated with methyl jasmonate and SA were less eaten by FAW [16]. These phytohormones may, in turn, be involved in the regulation of several downstream processes. Research on switchgrass revealed that FAW feeding increased the content of JA and its conjugates, terpenoids, and antifeedants. Moreover, the expression of terpenoids and phenylpropanoid pathway genes increased [17]. Among the antifeedants, the most common group of defensive compounds are terpenoids, phenolics, flavonoids, lignins, tannins and lipids. Moreover, several plant defensive proteins, e.g., protein inhibitors, lipoxygenases, peroxidases, catalases, superoxide dismutase, etc., are activated to protect the plant against herbivory [18]. 

However, how the Chinese cultivars respond to FAW herbivory is not well understood. In order to develop long-term strategies to control FAW, it is essential to understand the key pathways that are differentially regulated in maize plants. Which antifeedants are produced in maize plants and how their content can be increased needs continued exploration. One of the many strategies in this regard is the use of bamboo charcoal (BC). When applied, it mediates the increased biosynthesis of secondary metabolite. For example, in tomato, the application of BC increased plant growth and development and reduced the survival of the American tomato pinworm [19]. Bamboo charcoal has several advantages, such as being cost-effective, environmentally friendly, improving plant biomass and photosynthetic efficiency [20], having anti-herbivory activity and increased plant tolerance to insect pests [21]. 

To test if growing maize plants in BC can better perform under FAW herbivory, we tested three different combinations of BC with coconut bran against control and evaluated plant growth and chlorophyll content 10 and 20 days after sowing (DAS). We also studied the survival probability and larval and pupal survival of FAW on maize leaves grown in BC. Furthermore, we used comparative metabolomics and transcriptomics approaches to understand the key pathways that were differentially regulated when maize plants were grown in BC under FAW herbivory. We discuss global anti-herbivory responses in maize grown in BC under FAW infestation. 

## 2. Material and Methods

### 2.1. Plant Growth, Physiological Evaluation, and S. frugiperda Survival

Maize variety Denghai 605 was used as plant material. Seeds were disinfected by the method described earlier [22]. Plants were grown in square plastic flowerpots (7 cm wide and 9 cm high) filled with four biochar treatments, i.e., 10:1 (BC1), 30:1 (BC2), 50:1 (BC3), and pure coconut bran (CK) to BCcoal (*v*/*v*). The nutritional composition of the BC is given in Table S1. To promote plant growth, 2 g of large element water-soluble fertilizer (OMEX, 18-18-18) was applied per pot. Plants were placed in an insect-free glass greenhouse and watered once every two days. The greenhouse temperature and humidity were maintained at 24 ± 1 °C (day) and 20 ± 1 °C (night), and 60 ± 5%, respectively. During the experimental period, no pesticides were used in the greenhouse. Physiological indicators such as plant height (27 repetitions), stem diameter (27 repetitions), leaf dry-to-fresh weight ratio (5 repetitions), and chlorophyll content (5 repetitions) were measured at 10 and 20 DAS. Chlorophyll contents were measured according to a previously reported method in [23].

*Spodoptera frugiperda* was collected from Lishui, Zhejiang, in May 2019 and continuously raised in an artificial climate chamber (25 ± 1 °C, RH 60 ± 5%, 16L: 8D) using maize plants. One newly hatched armyworm larva was placed into a culture dish with a diameter of 9 cm and fed continuously for 12 days using various treatments of corn leaves that had grown to the 5-leaf stage, replacing fresh leaves every day. Probability of survival (%), larval survival (%) and pupal survival (%) were recorded for the three BC treatments and CK at five-leaf-stage maize plants. Each experimental group was repeated 12 times, with 5 larvae treated in each repetition. The BC treatment with the significantly lower survival % was further selected to compare transcriptome and metabolome profiles of leaves of corn plants. Five-leaf maize plants were selected, and the third leaf from each treatment was collected and immediately frozen in liquid nitrogen, then stored at −80 °C.

### 2.2. Metabolome Profiling 

#### 2.2.1. Sample Preparation and Extraction

Metabolites were extracted from leaves, with six biological replicates for both the plants grown on BC and CK. Samples were lyophilized in a lyophilizer (Scientz-100F, Ningbo Scientz Biotechnology Co., Ltd., Ningbo, China) and ground using a grinder (MM400, Retsch, Retsch GmbH, Haan, Germany) at 30 Hz for 1.5 min. Then, 50 mg of the sample powder was extracted with 1200 µL of pre-cooled (−20 °C) 70% methanol and vortexed for 30 s every 30 min for a total of six times. The extracts were then centrifuged at 12,000 rpm for 3 min, the supernatant was aspirated, and the samples were filtered through a microfilter (0.22 μm pore size) and stored in the injection vial for analysis. The data were analyzed using SPSS V 29 (https://www.ibm.com/products/spss-statistics; accessed on 11 June 2024). 

#### 2.2.2. UPLC Conditions

The six extracts were analyzed on a UPLC-ESI-MS/MS system (UPLC, ExionLC™ AD, MA, USA; https://sciex.com.cn/accessed on 14 February 2024) and a tandem mass spectrometry system (https://sciex.com.cn/, accessed on 14 February 2024). The analytical conditions were as described below.

For UPLC, we used an Agilent SB-C18 (Agilent, MA, USA) column (1.8 µm, 2.1 mm × 100 mm). The mobile phase consisted of solvent A, pure water with 0.1% formic acid, and solvent B, acetonitrile with 0.1% formic acid. Samples were measured using a gradient program. The starting conditions were 95% A and 5% B; within nine minutes, a linear gradient to 5% A and 95% B was automated, followed by maintaining a composition of 5% A and 95% B for one minute. Next, within 1.1 min, we adjusted the composition to 95% A and 5.0% B and kept it for 2.9 min. The flow velocity, column oven temperature, and injection volume were set to 0.35 mL/minute, 40 °C, and 2 μL, respectively. The effluent was alternatively connected to an ESI triple quadrupole-linear ion trap (QTRAP)-MS (SCIEX, MA, USA).

For ESI-Q TRAP-MS/MS, the ESI source operating parameters were as follows: source temperature 500 °C; ion spray voltage 5500 V (positive ion mode)/−4500 V (negative ion mode); ion source gas I, gas II and curtain gas were set to 50, 60 and 25 psi, respectively. Collision-activated dissociation was high. QQQ scans were acquired as MRM experiments with collision gas (nitrogen) set to medium. Declustering potential and collision energy for individual MRM transitions were performed with further optimization. A specific set of MRM transitions was monitored for each period according to the metabolites eluted within that time period.

#### 2.2.3. Metabolome Data Analysis

The metabolites were identified from Metware’s dedicated metabolite database (Metware Biotechnology Co., Ltd., Wuhan, China) in conjunction with different local metabolic databases: MzCloud, Massbank, Metlin, and HMDB by comparing the accurate precursor ion (Q1) and production (Q3) values, retention time and fragmentation pattern. After normalizing the original peak area information with the total peak area, the qualitative and quantitative aspects of metabolites were followed by searching the internal database and public databases. Unsupervised principal component analysis (PCA), Pearson’s correlation coefficient (PCC) and hierarchical cluster analysis were performed in R using prcomp, cor function and ComplexHeatmap packages, respectively (www.r-project.org, accessed on 14 February 2024). Differentially accumulated metabolites (DAMs) were screened using two criteria, i.e., variable importance in projection (VIP) > 1 and absolute Log2FC (|Log2FC| ≥ 1.0).

The identified metabolites were annotated and mapped in the KEGG Compound database (http://www.kegg.jp/kegg/compound/, accessed on 14 February 2024) and the KEGG Pathway database (http://www.kegg.jp/kegg/pathway.html, accessed on 14 February 2024). The pathways with significant DAM mapped to them were then fed into the metabolite set enrichment analysis, and their significance was determined by the hypergeometric test’s *p*-values.

### 2.3. Transcriptome Sequencing

#### 2.3.1. RNA Extraction, Library Construction, and Sequencing

RNA was isolated from the six samples using a Plant RNA Kit (BioTeke, Beijing, China). The quality of the RNA was checked using a nanophotometer spectrophotometer (ThermoFisher Scientific Inc., Waltham, MA, USA) as well as a Qubit 4.0 fluorometer (ThermoFisher Scientific Inc., Waltham, MA, USA). mRNAs were obtained from the total RNA, and cDNA was synthesized as reported earlier [24]. dscDNA was end-repaired, A-tailed and connected to sequencing adapters. Magnetic beads were then used to purify the DNA, followed by fragment size selection and PCR enrichment. Libraries were preliminarily quantified using the Qubit dye method, followed by the Q-PCR method to accurately quantify the effective concentrations (>2 nM). Libraries were then sequenced on an Illumina platform (Illumina Inc., San Diego, CA, USA).

#### 2.3.2. Transcriptome Data Analysis

Raw reads were checked for quality by using fastp [25], followed by the determination of error rate and GC content distribution. HISAT2 was used to align the clean reads with the reference genome (*Zea mays* cultivar B73) [26]. Gene expression was computed as Fragments Per Kilobase of transcript per Million fragments mapped (FPKM) using featureCounts [27]. Pearson’s correlation coefficient and PCA were computed in R. DESeq2 [28] was used to find differentially expressed genes (DEGs). Next, we used the Benjamini–Hochberg method to obtain the False Discovery Rate (FDR). The DEGs were screened based on the criteria of |log 2-Fold Change| ≥ 1 and FDR < 0.05. Cluster analysis on the DEG data was performed, and heatmaps were generated. The DEGs were functionally annotated in the KEGG and GO databases, and pathway enrichment was performed. Heatmaps for selected genes on specific pathways were generated using TBtools JRE1.6 [29].

### 2.4. Quantitative Real-Time PCR Analysis

Total RNAs were extracted using a modified CTAB method [30]. The cDNA was synthesized by using the MonScrip^TM^ first-strand cDNA Synthesis Kit (Monad Biotech Co., Ltd., Xuhui, Shanghai, China) according to the manufacturer’s instructions. PRIMER-BLAST [31] was used to design primers (Table S2). ZJBio qPCR SYBR Green Master Mix was used to conduct real-time fluorescence quantitative PCR using Applied Biosystems^TM^ ABI7500 quantitative PCR equipment (ThermoFisher Scientific Inc., Waltham, MA, USA). The *Actin* gene [32] was used as an internal control. Triplicate reactions were run for each gene. The relative gene expression was calculated using the 2^−∆∆CT^ formula [33].

## 3. Results

### 3.1. Effect of Biochar on Physiological Performance of Corn and Survival of S. frugiperda

**Figure 1:**
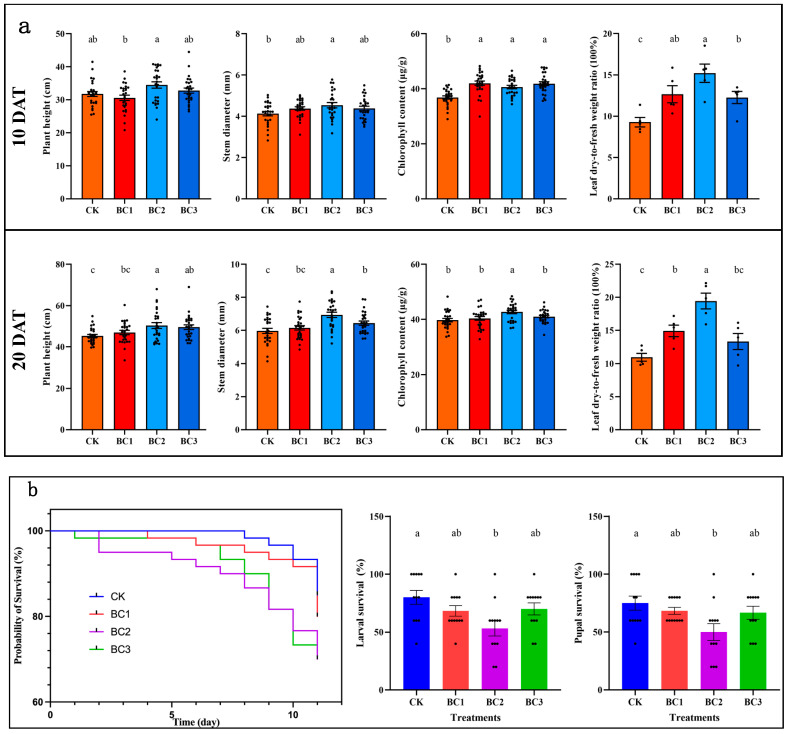
(**a**) Growth performance of maize in different biochar treatments 10 and 20 days after sowing. The bars show mean ± SEM (n = 27). (**b**) Probability of survival (%) of *S. frugiperda,* larval survival (%) and pupal survival (%). CK = control; and BC1, BC2 and BC3 are BCcoal to pure coconut bran (*v*/*v*) ratios, respectively. Bars on the plots show ± standard deviation (n = 60). The different letters on the bars indicate that the treatments differ significantly at *p* < 0.05. The bars show mean ± SEM (n = 12).

The effect of growing maize in different BC ratios is shown in Figure 1. Plants grown in BC2 had significantly higher plant height and stem diameter compared to BC1, BC3 and CK, both at 10 and 20 DAS. Similarly, a significantly higher leaf dry-to-fresh weight ratio was observed both at 10 and 20 DAS when plants were grown in BC2. The chlorophyll content of all BC treatments was significantly higher than CK, indicating a positive effect on plant growth. Though all the treatments showed similar content at 10 DAS, BC2 showed a pronounced increase in chlorophyll content at 20 DAS (Figure 1a). The probability of survival (%) was lowest for BC2 and BC3 after 10 days of infection. Larval survival (%) and pupal survival (%) were significantly lower in BC2 compared to CK, as well as BC1 and BC3 (Figure 1b). Based on the physiological parameters and survival (%), we selected BC2-grown leaves for omics analysis.

### 3.2. Metabolomes of S. frugiperda-Infested Maize Leaves under the Influence of Biochar

#### 3.2.1. Global Metabolome Profiles

**Figure 2:**
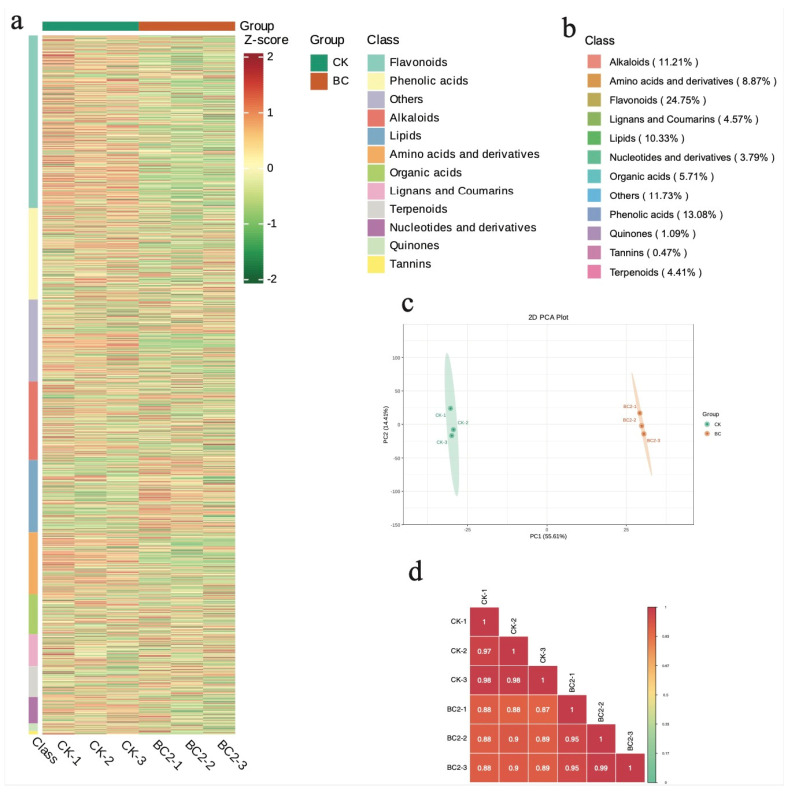
Global metabolome profile of maize leaves grown in BC under FAW herbivory. (**a**) Heatmap of metabolites detected in BC and CK. (**b**) The % of compounds in each class detected in BC and CK. (**c**) Principal component analysis and (**d**) Pearson’s correlation coefficient analysis of BC and CK based on relative metabolite intensities. BC = 30:1 (*v*/*v*) bamboo charcoal and coconut bran supplementation, and CK is without BC. Numbers (1–3) with BC and CK represent replicates.

Metabolomic profiling of six samples identified 1927 metabolites grouped into 12 classes (Figure 2a). The maximum number of compounds detected were flavonoids (24.75%), followed by phenolic acids (13.08%), alkaloids (11.21%) and lipids (10.33%) (Figure 2b). Both the grouping of sample replicates in PCA analysis and the higher PCC > 0.88 indicate the reliability of the replicates (Figure 2b,c).

#### 3.2.2. Differential Metabolome Profiles of BC and CK

**Figure 3:**
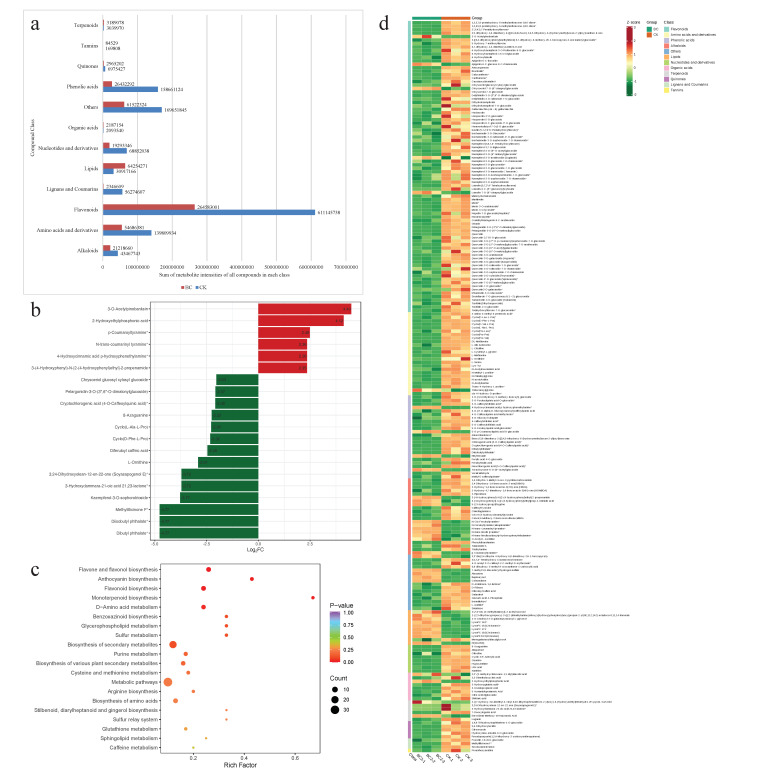
Differential metabolome profile of maize leaves grown in BC under FAW herbivory. (**a**) Sum of metabolite intensities of different compound classes in BC and CK. (**b**) Top up- and down-accumulated metabolites accumulated in BC vs. CK. (**c**) Scatter plot of KEGG pathway enrichment of differentially accumulated metabolites. (**d**) Heatmap of differentially accumulated metabolites in BC vs. CK. BC = 30:1 (*v*/*v*) bamboo charcoal and coconut bran supplementation, and CK is without BC. Numbers (1–3) with BC and CK represent replicates.

The differential analysis of metabolites led to the identification of 211 DAMs. As noted for the global metabolomic profiles, flavonoids were the top differentially accumulated class (84), followed by phenolic acid (24), amino acids and derivatives (24), and alkaloids (22). Flavonoids included anthocyanins, aurones, chalcones, flavonones, flavanols, flavones, flavonols, and other flavonoids. The cumulative metabolite intensity of each class showed that the contents of terpenoids, organic acids and lipids were higher in corn leaves grown in BC compared to CK (Figure 3a), whereas all the other compound classes showed a lower content in BC compared to CK. Top up-accumulated metabolites in BC included 3-o-acetylpinobanksin (flavonol), 2-hydroxyethylphosphonic acid (organic acid), p-coumaroyltyramine, N-trans-coumarinyl tyramine (alkaloids), and 4-Hydroxycinnamic acid p-hydroxyphenethylamine (phenolic acid). On the contrary, highly down-accumulated metabolites included diisobutyl phthalate, dibutryl phthalate (phenolic acids), methylillicinone F (coumarin), kaempferol-3-O-sophorotrioside (flavonol), and 3-hydroxydammara-21-oic acid 21,23-lactone (triterpene) (Figure 3b).

To further increase our understanding, we specifically focused on the DAMs enriched in specific pathways related to flavonoids, lipids, terpenoids, amino acids, phenolic acids and alkaloids. In general, the DAMs were enriched in 48 KEGG pathways (Figure 3c). Regarding flavonoids, 12 DAMs were enriched in flavone and flavonol, flavonoid and anthocyanin biosynthesis pathways. Except for 3-O-acetylpinobanksin, all other metabolites were accumulated in higher quantities in CK, indicating a reduction in flavonoid content. A similar accumulation trend was noted for DAMs enriched in monoterpenoid biosynthesis; stilbenoid, diarylheptanoid and gingerol biosynthesis; and phenylpropanoid biosynthesis pathways. Moreover, 11 DAMs enriched in twelve KEGG pathways related to amino acid biosynthesis also showed reduced content in BC-grown leaves (Figure 3d).

Nine of the ten lipids had increased accumulation in BC compared to CK, indicating a beneficial effect of biochar. These compounds include two glycerol esters (PI(18:2/0:0) and 1-O-linoleoyl-3-O-galactopyranosyl-L-glycerol), a free fatty acid (1′,2′,3′-tris-(3-methylbutanoyl)-2-acetyl sucrose), four lysophosphatidylcholines (LPCs) and two lysophosphatidylethanolamines (LPEs). As for alkaloids, three subclasses, i.e., isoquinoline (one compound), pyrrole (one compound) and phenolamines (six compounds), showed a significant increase in their content when maize was grown in BC under the influence of *S. frugiperda* herbivory. Two other DAMs classified as alkaloids, i.e., 3-(4-hydroxyphenyl)-N-[2-(4-hydroxyphenyl)ethyl]-2-propenamide and O-acetyl-L-carnitine, also showed significant up-accumulation in BC compared to CK.

Although most organic acids were down-accumulated, we noticed an increase in the levels of 2,2′-(3-methylcyclohexane-1,1-diyl)diacetic acid and 2-hydroxyethylphosphonic acid in BC compared to CK. Similarly, two of the six terpenoids, i.e., 2-((7-hydroxy-3,8-dimethyl-4-vinyl-9,10-dihydrophenanthren-2-yl)oxy)-6-(hydroxymethyl)tetrahydro-2H-pyran-3,4,5-triol and ent-16beta-methoxy-19-kauranoic acid, were accumulated at higher levels in BC compared to CK. Four (4-hydroxycinnamic acid p-hydroxyphenethylamine, 5-O-p-coumaroylquinic acid O-glucoside, ethyl ferulate and torachrysone-8-O-(6″-acetyl)glucoside) of the twenty-four phenolic acids were also up-accumulated. Other notable results include the increased accumulation of alnustone, bupleurylnol, cohumulone, 7-methyl-5,8-dioxodecyl hydrogen sulfate, 3,3′-Bis(3,4-dihydro-4-hydroxy-6,8-dimethoxy-2H-1-benzopyran) and tridecanoylglycine. In addition to these observations, we also found that abscisic acid, JA and SA were present in higher levels in BC-grown maize leaves compared to those of CK (Figure S1).

These observations highlight that the levels of terpenoids, organic acids, lipids and some specific compounds, as stated above, are higher in BC-grown maize leaves compared to CK, while the other metabolites show reduced accumulation. These observations suggest two possible scenarios. First, BC manipulates the degradation of tannins, phenolic acids, nucleotides and derivatives, lignans, flavonoids, amino acids and alkaloids to manage resources for plant survival. Second, BC induces an increased accumulation of terpenoids, organic acids, lipids and some other metabolites as anti-herbivory responses.

### 3.3. Transcriptome Profiles of S. frugiperda-Infested Corn Leaves under the Influence of Biochar

#### 3.3.1. Global Transcriptome Profile of BC vs. CK

**Figure 4:**
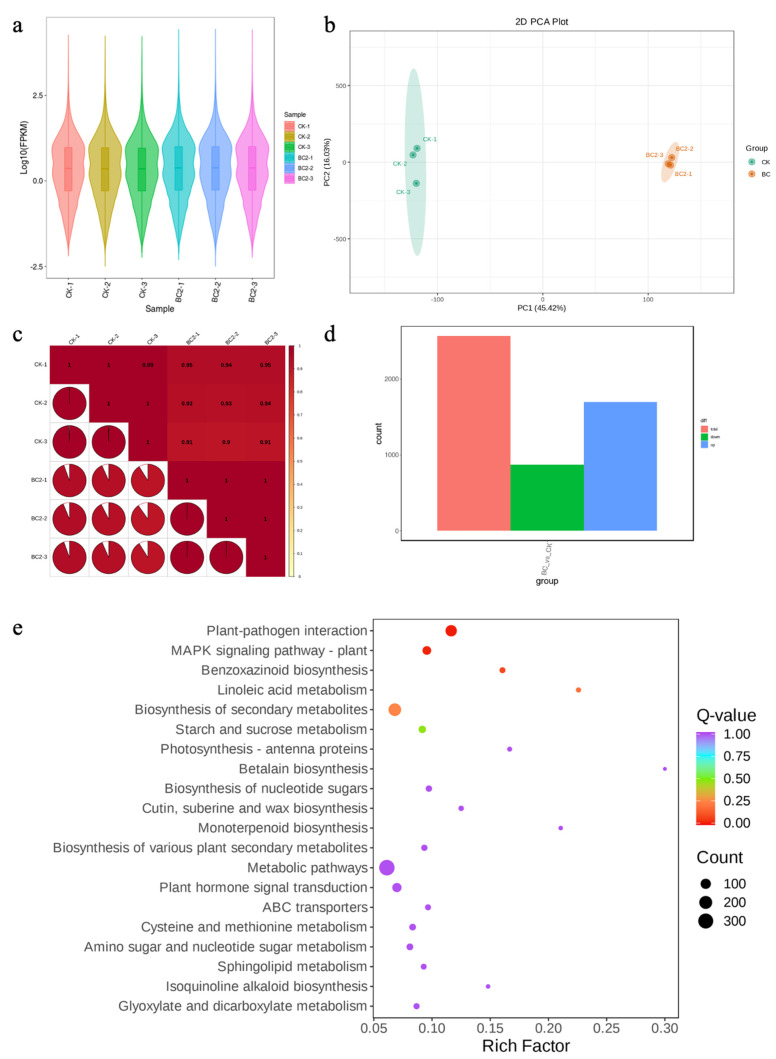
Global transcriptome profile of maize leaves grown in BC under FAW herbivory. (**a**) Overall distribution of gene expression, (**b**) principal component analysis and (**c**) Pearson’s correlation coefficient analysis based on gene expression. (**d**) Number of differentially expressed genes and (**e**) KEGG pathway enrichment scatter plot between BC and CK. BC = 30:1 (*v*/*v*) bamboo charcoal and coconut bran supplementation, and CK is without BC.

Illumina sequencing of six maize leaf cDNA libraries (three each for BC and CK) produced, on average, 58.5726 million raw and 56.93 million clean reads (~8 Gb), respectively, per library. The average error rate, GC content, Q20 and Q30 were 0.02%, 98.18%, 94.82% and 55.18%, respectively (Supplementary Table S2). More than 91.94% of the reads could be mapped to the reference genome. In total, 38,629 transcripts were expressed. The FPKM values of the BC samples were slightly higher than those of the CK (Figure 4a). The PCC (>0.90) and PCA indicated the reliability of the sampling (Figure 4b,c), similar to the results of metabolomic analysis.

#### 3.3.2. Differential Transcriptome Profile of BC vs. CK

A total of 2568 genes were differentially expressed between BC and CK; 1697 and 871 genes were up- and downregulated in BC compared to CK (Figure 4d). These DEGs were enriched in 131 KEGG pathways. Twenty KEGG pathways to which DEGs were significantly enriched are presented in Figure 4e. The top downregulated genes in BC were *novel.1721* (retrotransposon gag protein), *novel.1706* (plant transposase), *novel.3210* (*AdoHcyase*, adenosylhomocysteinase), *Zm00001eb201380* (phosphoenolpyruvate carboxykinase (ATP)) and *Zm00001eb033760* (oligopeptide transporter 5). The most upregulated genes in BC were *novel.5234* (protein TIF31), *Zm00001eb369760* (pre-mRNA-processing factor 39), *Zm00001eb172840* (E3 ubiquitin-protein ligase FANCL), *novel.1277* (FAR-RED IMPAIRED RESPONSE 1) and *Zm00001eb429390* (alcohol dehydrogenase (NADP+)) (Table S4). Of the 10 BC-specific genes, 6 were annotated. These included isocitryate lyase, nuclear pore complex protein Nup98-Nup96, alcohol dehydrogenase, E3 ubiquitin-protein ligase FANCL, pre-mRNA-processing factor 39 and protein TIF31 (Table S2). These observations indicate that BC induces several pathways in maize leaves under FAW herbivory.

To explore the transcriptome data in detail, we specifically focused on the pathways associated with the observations of metabolome results, transcription factors, defense and signaling-related pathways.

- Expression changes related to observed metabolome profiles

Differential BC vs. CK metabolome profiles showed changes in the accumulation of flavonoids, anthocyanins, amino acids, terpenoids, phenolic acids, alkaloids, tannins, nucleotides and derivatives, organic acids, lipids and specific metabolites. Therefore, we focused on the DEGs enriched in these metabolites related to KEGG pathways. As for the flavonoid biosynthesis pathway, 13 transcripts associated with eight genes were differentially expressed. The downregulation of the transcripts annotated as chalcone isomerase, flavonoid 3′-monooxygenase (*CYP75B1*), shikimate O-hydroxycinnamoyltransferase, and flavonol-3-O-L-rhamnoside-7-O-glucosyltransferase (UGT73C6) in BC is consistent with the reduction of flavonoid content. On the other hand, the upregulation of anthocyanidin reductase (ref. [34] is reported to increase cassava resistance to the two-spotted spider mite). Since we noted reduction of isoquercitrin (a flavonol) in metabolome results, the increased expression of flavonol-3-O-glucoside L-rhamnosyltransferase in BC is consistent. These changes indicate that a reduction in the expression of flavonoid biosynthesis genes causes reduced flavonoid accumulation in BC under FAW herbivory (Figure 4; Table S4).

As for amino acid (and derivatives) biosynthesis, 29 transcripts were enriched in arginine biosynthesis, alanine, aspartate and glutamate metabolism, biosynthesis of amino acids, valine, leucine and isoleucine biosynthesis, and cyanoamino acid metabolism. Notably, GLUTAMATE DUMPER 4, aspartate kinase, phosphoglycerate kinase, beta-glucosidase, tryptophan synthase, glutamine synthetase, folypolyglutamate synthase, delta-1-pyrroline-5-carboxylate synthetase and others were downregulated in BC. These observations are consistent with reduced amino acid and derivatives biosynthesis. However, several genes were also upregulated in BC, including proline dehydrogenase, aspartate kinase, polyamine antiporter and glutathione-S-transferase 23 (*GST23*) (Figure 4; Table S4). These observations indicate increased amino acid conversion to other compounds or transport, whereas the GSTs have been reported to play important roles in plants’ responses to biotic stresses [35], as they are an important part of ROS homeostasis.

Terpenoid content increased in maize leaves grown under BC during *S. frugiperda* infestation. Nineteen transcripts (five down- and fourteen upregulated in BC) enriched in terpenoid backbone and mono-, di- and tri-terpenoid biosynthesis pathways. Notably, the upregulation of genes enriched in terpenoid backbone biosynthesis, i.e., hydroxymethylglutaryl-CoA reductase (NADPH) (*HMGCR*), ditrans,polycis-polyprenyl diphosphate synthase (*GA2ox*), and geranylgeranyl diphosphate synthase (*GGPS*), suggests that growing corn in BC may increase terpenoid backbone biosynthesis by upregulating these genes. This is similar in the downstream upregulation of mono-, di- and tri-terpenoid biosynthesis. For this, the increased expression of (-)-germacrene D synthase (*GERD*), achilleol B synthase (*OSC6*), napthoate synthase (*menB*), 4-coumarate—CoA ligase (*4CL*), NAD(P)H dehydrogenase (quinone) (*wrbA*), and gibberellin 2beta-dioxygenase, etc., suggest BC may affect the expression of several genes (Figure 4; Table S4). These results are consistent with the metabolome profiles and highlight that BC increases terpenoid biosynthesis, thus enabling corn to survive better when infested with *S. frugiperda.*

Among other metabolites, a notable increase in lipids was observed in corn plants grown under BC. Five genes (two 3-ketoacyl-CoA synthases, a long-chain acyl-CoA synthetase and two fatty acyl-ACP thioesterases) were upregulated in BC compared to CK. These genes lead to octanoic acid, decanoic acid, dodecanoic acid, tetradecanoic acid and hexadecanoyl-CoA biosynthesis. Hexadecanoyl-CoA is also then used for glycerolipid and glycerophospholipid metabolism, as well as fatty acid elongation. We noted the upregulation of genes enriched in glycerolipid metabolism, i.e., alcohol dehydrogenase (NADP+), glycerol-3-phosphate acyltransferase, 1-acyl-sn-glycerol-3-phosphate acyltransferase, diacylglycerol kinase (ATP) and phospholipase D1/2. Similarly, the expression of genes enriched in glycerophospholipid metabolism and sphingolipid metabolism in BC (Figure 4; Table S4) is consistent with the observed increased accumulation of lipids and glycerol esters. Thus, growing corn in BC increases lipid biosynthesis, which is a possible deterrent to *S. frugiperda*.

b. Expression changes in defense-related genes

**Figure 5:**
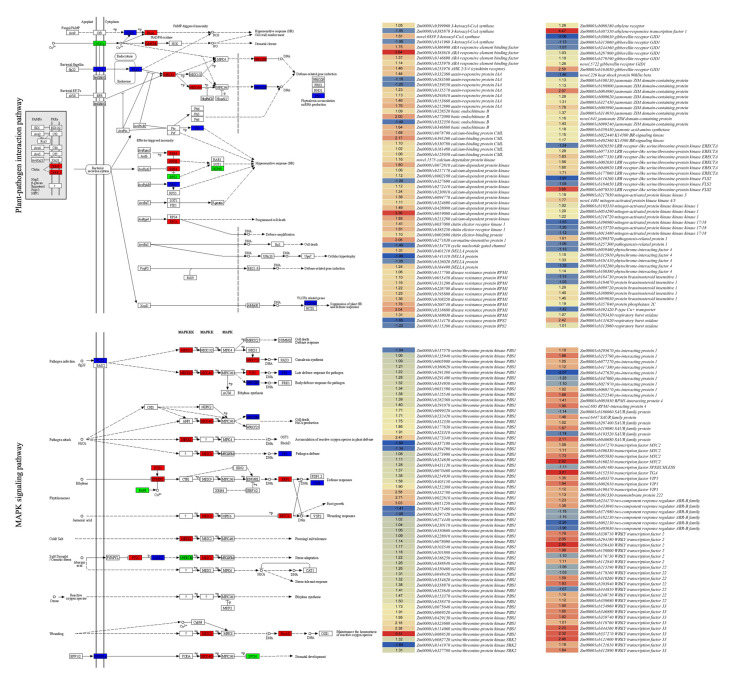
Differential regulation of plant–pathogen interaction and signaling pathways. Top panel shows plant–pathogen interaction KEGG pathway (04626), and bottom left panel shows MAPK signaling—plant KEGG pathway (04016). Heatmaps show log 2-foldchange values of genes enriched in plant–pathogen interaction and signaling pathways (MAPK and phytohormone). Heatmaps were prepared in TBtools [29].

Plants under biotic stress activate defense mechanisms to protect themselves and deter the invading pests [36]. We screened genes that were enriched in plant–pathogen interaction pathways (137 DEGs). Interestingly, BC-grown maize leaves had reduced expression of a cyclic nucleotide gated channel, indicating changes in Ca^2+^ transport. Moreover, several downstream genes in this reaction, i.e., calcium-dependent protein kinase (*CDPK*), mitogen-activated protein kinase kinase 4/5 (*MAPKK4/5*), mitogen-activated protein kinase kinase kinase 1 (*MEKK1*), WKRY33, WRKY22 and respiratory burst oxidase (*Rboh*), were upregulated in BC. These suggest BC improves maize resistance by activating ROS homeostasis-related genes, which ultimately induce the expression of defense-related genes, e.g., pathogenesis-related protein 1 (PR1). Several genes related to hypersensitive response (HR) were also differentially expressed, including RPM1-interactin protein 4 (*RIN4*), disease resistance protein 1 (*RPM1*), chitin elicitor-binding protein (*CEBiP*) and serione/threonine-protein kinase (*PBS1*). On the other hand, *RPS2* was downregulated, which is consistent with *RIN4* upregulation (Figure 5; Table S4). It is known from previous work that *RIN4* negatively regulates *RPS2* [37]. These observations highlight that BC-grown corn leaves resist *S. frugiperda* better than CK because of the increased expression of the plant–pathogen interaction-related genes.

c. Expression changes in phytohormone and MAPK signaling pathways

Seventy-two and fifty-eight DEGs were enriched in plant hormone signal transduction and the MAPK signaling pathway, respectively. Among the hormone-signaling genes, auxin and gibberellin-related genes showed mixed expression trends (some transcripts were upregulated, while the others were downregulated). Most importantly, the genes related to abscisic acid, ethylene, brassinosteroid, JA, and SA signaling were mostly upregulated in BC. The upregulation of 2C-type protein phosphatases (*PP2C*) and abscisic acid (ABA)-responsive element binding factor and the downregulation of some of the SNF1-related protein kinases2 (*SnRK*) indicate that BC improves ABA signaling. The upregulation of JA signaling genes, i.e., *JAR1*, *COI1*, *JAZ* and *MYC2*, and increased JA accumulation in BC (1.5-fold) suggested BC induces JA biosynthesis and signaling, which is present upstream of terpenoid biosynthesis. As we observed the increased expression of several PR1 genes (see above section), this might also be related to higher expression of transcription factor *TGA*, which is a part of SA signaling (Figure 5; Table S4). This is also consistent with the increased SA accumulation in BC.

Most of the genes, such as WRKYs, *Rboh*, *PR1*, *PP2C*, *SnRK*, *MAPKK4/5* and *MEK1,* were enriched in plant–pathogen interaction and plant hormone signaling pathways and were common with the MAPK signaling pathway. In addition to these, we observed the increased expression of transcription factor *VIP1*, LRR receptor-like serine/threonine-protein kinase ERECTA, basic endochitinase B and transmembrane protein 222 in BC (Figure 5; Table S4).

Taken together, these expression changes indicate BC improves anti-herbivory by regulating phytohormone and MAPK signaling pathways.

d. Expression changes in transcription factors

Transcription factors play essential roles in defense roles in plants against pest infestation. There were 88 differentially expressed TFs between CK and BC. These TFs belonged to 17 classes. The highest number of TFs were classified as WRKYs (24), followed by *bHLH* (10), *AP2/ERF-ERF* (9) and *tify* (8) (Figure S2). A total of 69 TFs were upregulated in BC compared to CK. Phytochrome-interacting factor 4, *C2C2-LSD* and the two-component response regulator ARR-A family were downregulated in BC compared to CK. Most of these TFs were enriched in plant hormone, MAPK signaling, and plant–pathogen interaction pathways (Table S4). Thus, the upregulation of these TFs further confirms that BC plays a role in activating these pathways and enabling corn to generate anti-herbivory responses.

**Figure 6:**
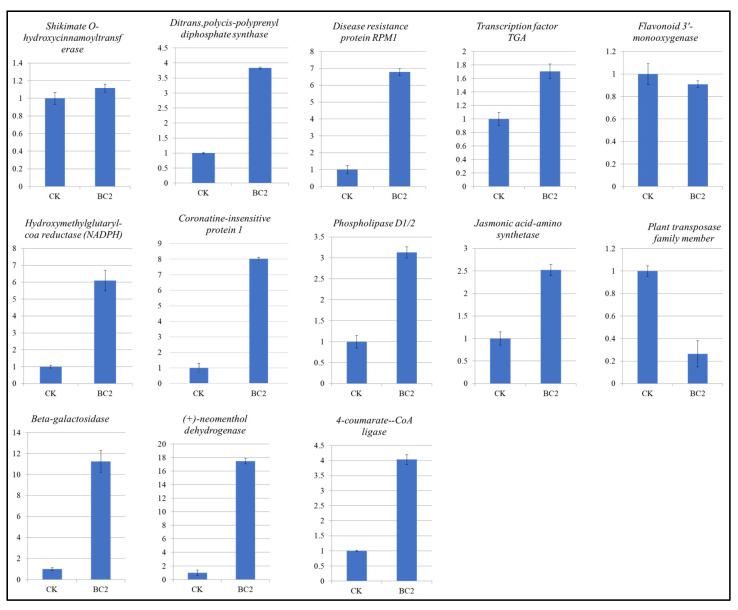
Quantitative real-time PCR analysis of maize genes in CK and BC2 leaves infested with *S. frugiperda.* The bars represent relative gene expression values (mean of *n* = 3). The error bars represent ± standard deviation.

For further validation of RNA sequencing results, we analyzed the expression of 13 maize genes by qRT-PCR (Figure 6). The expressions were consistent with the transcriptome sequencing results, indicating the reliability of sequencing. Notably, the upregulation of RPM1 protein 1, TGA TF, flavonoid 3′-monoxygenases, 4CL, phospholipase D1/2, JAR1, etc. confirms that HR is activated and the biosynthesis of terpenoids, flavonoids, phospholipids and JA is affected when BC is used (Figure 6).

## 4. Discussion

### 4.1. Bamboo Charcoal Improves Maize Plant Growth and Decreases FAW Survival

Biochar supplementation of soil or plant growth medium has shown positive effects on plant growth and development [38]. With the increasing concerns about the spread of FAW in China, the use of BC in maize could help farmers reduce the losses to herbivory. Our results that 30:1 (BC2) compared to BC1 and BC3 showed significantly better results both at 10 and 20 DAS are very valuable (Figure 1). Our data suggest a growth-promoting effect on maize and an anti-herbivory effect on FAW. These results are consistent with an earlier study, where the application of a 30:1 (*v*/*v*) ratio of BCcoal and coconut bran yielded maximum chlorophyll content, plant height and stem thickness in tomato [19]. Thus, the large-scale usage of BC can be a possible strategy. Such efforts have been reported from other countries. For example, researchers in Indonesia tested several doses of BC on the growth of maize in dry land and recommended its usage on a large scale [39]. Our results that FAW survival %, larvae and pupae survival % decreased in maize grown in BC2 suggest that BC2 not only improves growth and development but also improves anti-herbivory ability. Similar results on the improvement of the anti-herbivory ability of tomato against *Tuta absoluta* [19] and *Meloidogyne incognita* [40,41], rice against *Sogatella furcifera* [42] and others have been reported. Therefore, our results indicate that the application of BC improves plant growth and development, as well as the anti-herbivory ability of maize plants.

### 4.2. Bamboo Charcoal Induces Phytohormone and MAPK Signaling and Defense Responses in Maize Leaves against FAW Herbivory

Plants defend themselves against herbivores by direct defenses, such as ROS homeostasis, secondary metabolite biosynthesis, resistance proteins and phytohormone biosynthesis and signaling [18,36]. Herbivory induces differential calcium responses in plants and Ca^2+^ concentration changes in the cytosol, which triggers ROS [43]. The increased expression of *Rboh* and *CDPK* indicates that BC modulates Ca^2+^. *CDPKs* propagate immune signals required for resistance against biotic stress [44], which is evident from the increased expression of WRKYs (*WRKY22* and *WRKY33*) (Table S4). WRKYs are both part of the plant defense system against herbivory and act as part of the signaling cascade to activate the expression of genes associated with defense responses [45]. This is evident from the increased expression of the PR1 gene in BC-grown maize leaves under the FAW herbivory, which is consistent with the earlier work where PR protein expression increased in maize under FAW attack [46]. PR1 proteins are usually abundant in the apoplast during plant–pathogen interaction and inhibit pathogens [47]. PR1 proteins are induced as a response to wounding due to insects or changes in JA. These play anti-herbivory roles in plants, e.g., tomato [48]. Furthermore, increased *MAPKK4/5* and *MEKK1* expressions in BC (Figure 5) are consistent with earlier reports that WRKY TFs modulate plant immunity against insects (white fly) by interacting with the MAPK signaling cascade [49]. Plants differ in their defense responses against herbivores [50]. Some plants use HR to protect themselves against invading herbivores, e.g., soybean when attacked by *S. frugiperda* [51]. Our data also suggest that BC increasingly activated the HR, as evident from the higher expression of several genes, i.e., *RPM1*, *CEBiP*, *PBS1* and the downregulation of *RPS2* (Figure 5). Thus, we conclude that BC improves maize tolerance to FAW through the use of HR, ROS homeostasis, and Ca^2+^ signaling.

In addition to calcium influx and HR, herbivory also induces phytohormone and MAPK signaling cascades in plants [52]. Several herbivore insect species have been reported to induce phytohormone (notably ABA, JA and SA) biosynthesis and signaling, e.g., spider mites [53], *Spodoptera litura*, *Spodoptera exigua*, *Frankliniella occidentalis*, *Tetranychus urticae* and *Liriomyza sativa* [54]. The higher ABA levels and the increased expression of ABA-signaling genes under FAW herbivory suggest BC induces ABA-driven defense responses. Similar results have been reported earlier under *S. exigua* herbivory [55]. Additionally, jasmonates have previously been implicated in defense responses in tomato and maize foliage against *S. exigua* [56]. The increased JA levels in BC-grown maize leaves are also consistent with the higher terpenoid levels, indicating a possible quantitative relationship, as reported for *S. exigua* [57]. This was specifically evident for the BC-grown maize, suggesting that BC plays a role in increased JA biosynthesis and induces downstream signaling. Biochar is known to alter JA levels in plants [42]. Thus, our results suggest a similar effect of BC in maize leaves under FAW herbivory. We also detected higher levels of SA and the genes involved in signaling, i.e., *TGA* (Figure 5). Earlier work on maize has shown that SA positively regulates defense responses against lepidopteran insects [58]. Elevated SA levels have also been linked with increased defense signaling [59]. However, the cross-talk between JA and SA under FAW herbivory should be further investigated in specific experiments, considering earlier reports that SA can actually inhibit J-induced resistance to *S. exigua* in Arabidopsis [60]. Taken together, our combined metabolomic and transcriptomic data indicate that BC supplementation alters the endogenous phytohormone levels in maize leaves, which then induce changes in genes enriched in phytohormone and MAPK signaling pathways.

### 4.3. Bamboo Charcoal Induces Differential Regulation of Maize Leaf Secondary Metabolites against FAW Herbivory

To understand which pathways are being regulated as a result of BC supplementation in maize plants under FAW herbivory, we investigated the global metabolome and transcriptome responses in maize leaves under FAW herbivory. Earlier research on biochar-supplemented plants under different herbivore attacks has shown differential regulation of primary and secondary metabolites [61]. However, as different plant species may interact differently with herbivores [62,63], generalized metabolomic and/or transcriptome responses cannot be expected. In addition, plants may also induce herbivore-specific defense responses [64]. For example, it has been reported that soybean (and wild soybean) increases flavonoid biosynthesis in response to herbivory [65]. Similarly, several flavonoid biosynthesis genes were upregulated as a result of FAW herbivory in rice [11]. Normally, flavonoid biosynthesis is activated by the elicitation of insect herbivory [66], but BC supplementation is possibly counteracting this activation by reducing the expressions of related genes (Figure 4). Therefore, flavonoid levels did not increase in response to herbivory. The expression differences in several pathways related to flavonoid biosynthesis are consistent with the flavonoid levels, thus providing valuable preliminary data on the transcriptome and metabolome levels. These genes should be further characterized in gene knock-out studies. Similarly, the higher amino acid levels in CK leaves are consistent with previous reports that herbivory induces amino acid metabolism [67]. As amino acids are one of the major forms of nitrogen in plants, they play roles in plant survival and growth. Lower levels of amino acids in BC may indicate their degradation and use as precursors for the biosynthesis of defense compounds [66]. This proposition corresponds to the metabolomic profile of BC vs. CK and the increased expression of several genes enriched in valine, leucine and isoleucine degradation (KEGG pathway 00280). Thus, our data propose that FAW herbivory might induce increased flavonoid and amino acid biosynthesis and that BC supplementation activates the regulation of these pathways for resource management and anti-herbivory compound biosynthesis. This could also explain the higher terpenoid levels in BC (Figure 3). We say this because the terpenoid backbone biosynthesis pathway is present downstream of the valine, leucine and isoleucine degradation pathway [68].

Experiments have shown that the addition of biochar improves the concentration of terpenes, e.g., in basil [69] and tomato [70]. The increased expression of several genes involved in terpenoid backbone and terpenoid biosynthesis (Figure 4) further validates this proposition. The gene GGPS synthesizes geranyl diphosphate and is present upstream of the terpenoid biosynthesis pathways. Overexpression of *GGPS* has previously been reported to increase monomeric and dimeric terpenes in *Catharanthus* roseus [71]. Together with the higher expression of *GA2ox*, *HMGCR*, *GERD*, *OSC6*, *menB*, *4CL* and *wrbA*, the increased *GGPS* expression and the higher levels of the terpenoid class of compounds (particularly diterpenoids) suggests that BC triggers terpenoid biosynthesis (Figure 4; Tables S3 and S4). This increased terpenoid level and reduced survival % of FAW on BC-grown maize leaves is also consistent with the known role of these compounds. Terpenoids are known to mediate plant–insect interactions, mostly in favor of plants. When plants have higher terpenoid levels, herbivores cause less damage to the plant organs, as noted in the case of *Melaleuca alternifolia* against leaf beetles [72]. Apart from the terpenoids, our results also indicate that BC improves lipid/fatty acid levels (Table S3). Earlier studies in maize [73] and wheat [74] have also reported similar instances where biochar application improved lipid metabolism. This is consistent with the increased expression of several fatty acid, glycerolipid, glycerophospholipid and sphingolipid metabolism genes.

### 4.4. Bamboo Charcoal Regulates the Expression of a Large Number of Transcription Factors in Maize Leaf against FAW Herbivory

Transcription factors are master regulators of defense responses and signaling in plants against biotic and abiotic stresses [75]. Several classes of TFs have been implicated in anti-herbivory in plants, e.g., TFs differentially contribute to defense in Arabidopsis against *S. littoralis* [76]. Our results that 17 TF classes were differentially activated in maize leaves under FAW herbivory indicate BC induces regulation of several TF families (Table S4). Transcriptional regulation of inducible defenses in plants against herbivores is mostly centered on MAPK and phytohormone signaling, downstream pathways and the plant–pathogen interaction pathway [77]. The fact that 68 of the 88 TFs were enriched in these pathways indicates that BC plays a significant role in the transcriptional activation of these signaling and defense cascades. The increased expression of a large number of TFs involved in ABA signaling suggests that BC enhances the rapid induction of auxin-dependent defense responses in maize leaf against FAW herbivory. Thus, the upregulation of these TFs (e.g., the auxin-responsive protein IAA) further confirms the role of BC in activating these pathways and enabling maize to generate anti-herbivory responses [78]. Furthermore, the increased expression of JA signaling-related TFs (*tify*, *JAZ* and *MYC*) indicates that BC supplementation induces JA-dependent responses in maize leaves against FAW herbivory. This, together with the other phytohormone and MAPK signaling genes and defense-responsive genes discussed in the above sections, indicates BC is an inducer of increased anti-herbivory supplements. Similar observations have been reported in tea [79] and tobacco [80] in relation to the emission of secondary metabolites (volatiles) against herbivory. Other TFs whose expression BC increased in maize leaves under FAW herbivory include *bHLH*. This, in turn, is relevant for JA signaling. Indeed, *bHLH*, together with *JAZ* and *MYC2*, is a master regulator of JA-dependent defense responses [77]. Taken together, our results show that BC can induce expression changes in a large number of TFs belonging to different classes.

## 5. Conclusions

Maize plants were grown in three combinations of BCcoal and coconut bran (*v*/*v*), i.e., 10:1 (BC1), 30:1 (BC2) and 50:1 (BC3). BC2 had significantly higher plant height, stem diameter, chlorophyll (only DAS) content and leaf dry-to-fresh weight ratio at 10 and 20 DAS. Similarly, the probability of FAW and its larvae and pupae survival was lowest in BC2. Thus, we conclude that BC2 is the best supplement to improve the anti-herbivory of maize leaves. The combined metabolome and transcriptome analysis of the maize leaves grown in BC2 under FAW herbivory had lower flavonoids, amino acids and derivatives, while it had higher terpenoids (mainly diterpenoids) and lipid content. Similarly, BC-grown maize leaves had higher ABA, SA and JA content. The transcriptome profiles were consistent with the metabolome profiles. Our results imply that growing maize in BC improves its anti-herbivory by inducing changes in secondary metabolite pathways, hormone and MAPK signaling, and defense-related genes.

## Data Availability

The original contributions presented in the study are included in the article/Supplementary Material. The metabolome data have been submitted to the MetaboLights database, accessible at: https://www.ebi.ac.uk/metabolights/editor/MTBLS10975/descriptors.

## Supplementary Materials

The following supporting information can be downloaded at: https://www.mdpi.com/article/10.3390/metabo14090498/s1, Figure S1. Sum of metabolite intensities of compounds identified as abscisic acid, jasmonic acid and salicylic acid in BC and CK; Figure S2. Number of differentially expressed transcription factors in bamboo charcoal-grown maize leaves under *S. frugiperda* herbivory; Table S1. Composition of biochar and its properties; Table S2. List of primers used for qRT-PCR analysis of maize leaves; Table S3. Summary of Illumina sequencing of six corn-leaf cDNA libraries; Table S4. Expression changes in genes enriched in specific pathways in biochar-supplemented corn leaves infested with *S. frugiperda*.
