# Supplementary material for: Combined Metabolome and Transcriptome Analyses of Maize Leaves Reveal Global Effect of Biochar on Mechanisms Involved in Anti-Herbivory to Spodoptera frugiperda
Source: Metabolites. 2024 Sep 14;14(9):498. doi: 10.3390/metabo14090498 (PMC11433984; doi:10.3390/metabo14090498)
Supplement: Supplementary file 1 [file metabolites-14-00498-s001.zip › Figure S2.pptx]

## Slide 1
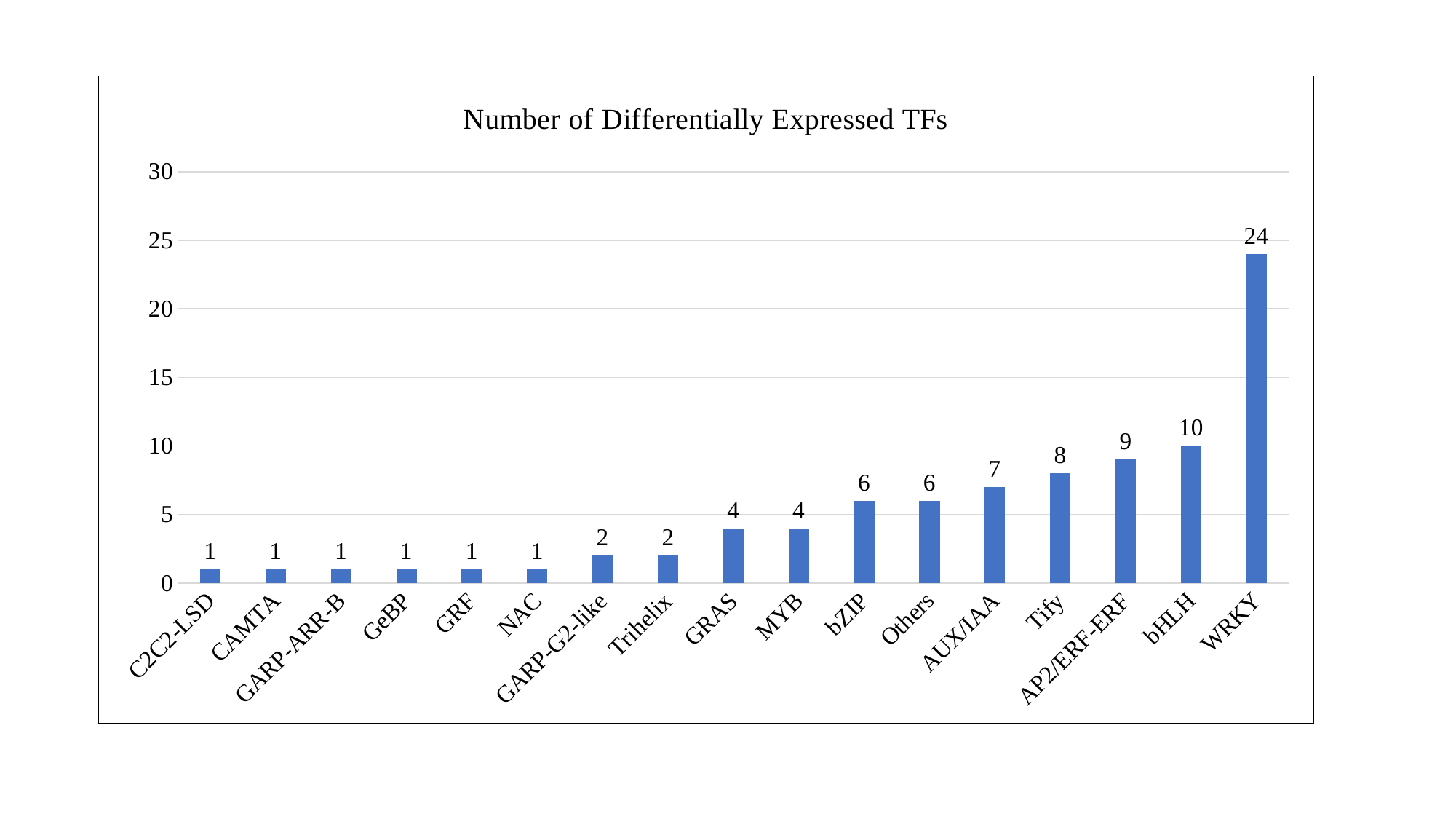

### Chart: Number of Differentially Expressed TFs
| Category | |
|---|---|
| C2C2-LSD | 1.0 |
| CAMTA | 1.0 |
| GARP-ARR-B | 1.0 |
| GeBP | 1.0 |
| GRF | 1.0 |
| NAC | 1.0 |
| GARP-G2-like | 2.0 |
| Trihelix | 2.0 |
| GRAS | 4.0 |
| MYB | 4.0 |
| bZIP | 6.0 |
| Others | 6.0 |
| AUX/IAA | 7.0 |
| Tify | 8.0 |
| AP2/ERF-ERF | 9.0 |
| bHLH | 10.0 |
| WRKY | 24.0 |
