# Supplementary material for: Combined Metabolome and Transcriptome Analyses of Maize Leaves Reveal Global Effect of Biochar on Mechanisms Involved in Anti-Herbivory to Spodoptera frugiperda
Source: Metabolites. 2024 Sep 14;14(9):498. doi: 10.3390/metabo14090498 (PMC11433984; doi:10.3390/metabo14090498)
Supplement: Supplementary file 1 [file metabolites-14-00498-s001.zip › R1 Figure S1.pptx]

## Slide 1
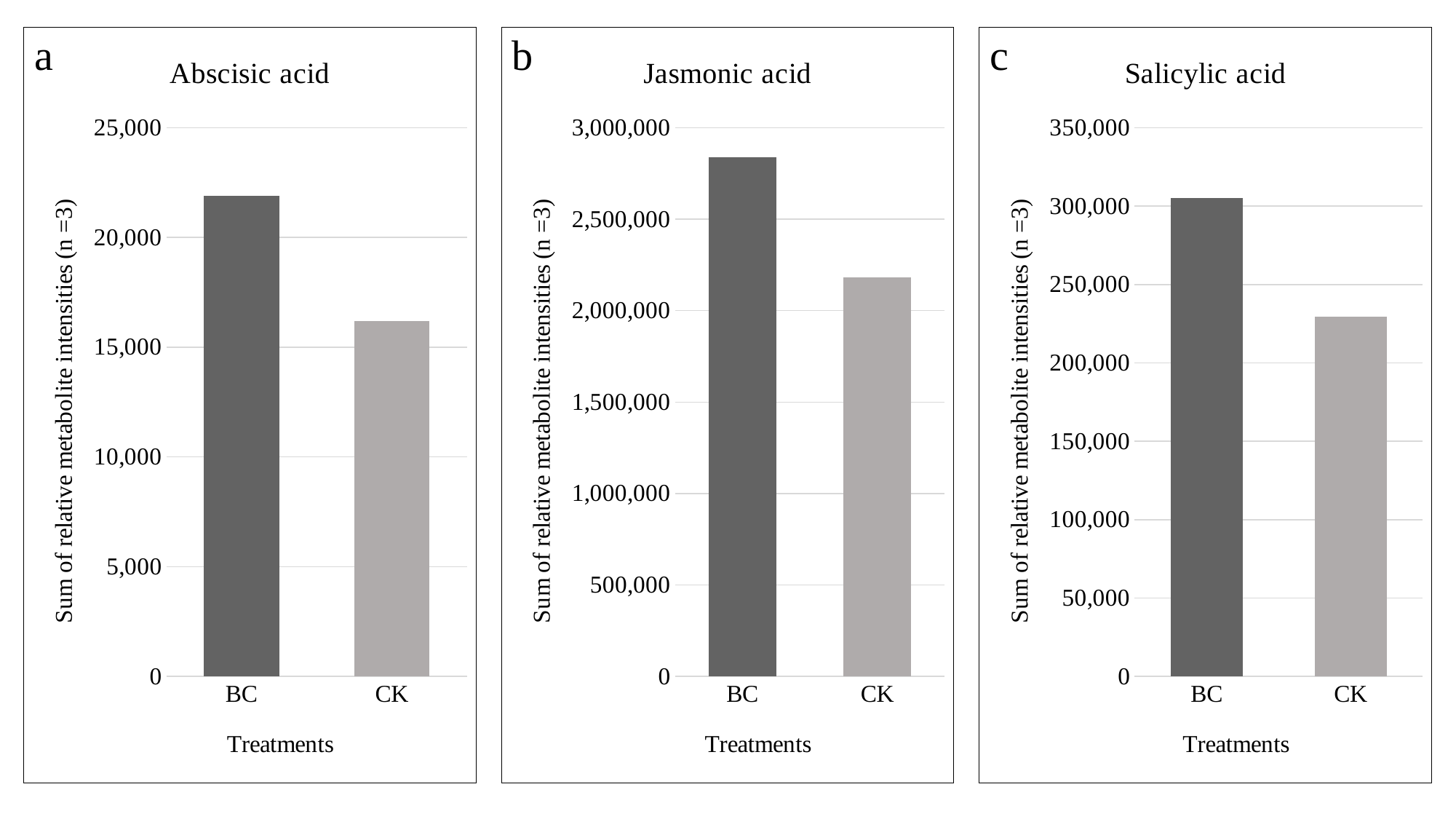

### Chart:
| Category | Abscisic acid |
|---|---|
| BC | 21898.41119 |
| CK | 16177.845916666667 |
### Chart:
| Category | Jasmonic acid |
|---|---|
| BC | 2838753.332 |
| CK | 2181867.7423333335 |
### Chart:
| Category | Salicylic acid |
|---|---|
| BC | 305175.6404 |
| CK | 229601.79216666668 |
